# Functional mapping of microRNA promoters with dCas9 fused to transcriptional regulators

**DOI:** 10.3389/fgene.2023.1147222

**Published:** 2023-05-05

**Authors:** Pradeep Kumar, Mathilde Courtes, Céline Lemmers, Anne Le Digarcher, Ilda Coku, Arnaud Monteil, Charles Hong, Annie Varrault, Runhua Liu, Lizhong Wang, Tristan Bouschet

**Affiliations:** ^1^ Department of Genetics, University of Alabama at Birmingham, Birmingham, AL, United States; ^2^ O’Neal Comprehensive Cancer Center, University of Alabama at Birmingham, Birmingham, AL, United States; ^3^ Institut de Génomique Fonctionnelle, CNRS, INSERM, Université de Montpellier, Montpellier, France; ^4^ Plateforme de Vectorologie de Montpellier (PVM), BioCampus Montpellier, CNRS, INSERM, Université de Montpellier, Montpellier, France; ^5^ Vanderbilt University School of Medicine Nashville, Nashville, TN, United States

**Keywords:** microRNA, CRISPRa, CRISPRi, promoter, *Mest*/PEG1, miR-335, miR-3662, embryonic stem (ES) cell

## Abstract

MicroRNAs are small non-coding RNAs that control gene expression during development, physiology, and disease. Transcription is a key factor in microRNA abundance and tissue-specific expression. Many databases predict the location of microRNA transcription start sites and promoters. However, these candidate regions require functional validation. Here, dCas9 fused to transcriptional activators or repressors - CRISPR activation (CRISPRa) and inhibition (CRISPRi)- were targeted to the candidate promoters of two intronic microRNAs, mmu-miR-335 and hsa-miR-3662, including the promoters of their respective host genes Mest and HBS1L. We report that in mouse embryonic stem cells and brain organoids, miR-335 was downregulated upon CRISPRi of its host gene Mest. Reciprocally, CRISPRa of Mest promoter upregulated miR-335. By contrast, CRISPRa of the predicted miR-335-specific promoter (located in an intron of Mest) did not affect miR-335 levels. Thus, the expression of miR-335 only depends on the promoter activity of its host gene Mest. By contrast, miR-3662 was CRISPR activatable both by the promoter of its host gene HBS1L and an intronic sequence in HEK-293T cells. Thus, CRISPRa and CRISPRi are powerful tools to evaluate the relevance of endogenous regulatory sequences involved in microRNA transcription in defined cell types.

## Introduction

microRNAs (miRNAs) are short non-coding RNAs that play a central role in regulating gene expression in plants and animals (Jones-Rhoades et al., 2006; Bartel, 2018). miRNAs impact development and physiology and are dysregulated in diseases, including cancer (Schanen and Li, 2011; DeVeale et al., 2021; Xue et al., 2021). Stringent gene annotations suggest that there are ∼500 miRNAs in mice (Chiang et al., 2010) and humans (Fromm et al., 2015). It is estimated that approximately half of the mammalian miRNAs are intronic (Rodriguez et al., 2004; Meunier et al., 2013; Hinske et al., 2014). miRNA biogenesis sequentially involves transcription, cleavage of the miRNA hairpin precursor out of the primary transcript, transport of intermediate forms, and loading of the mature miRNA into the RNA-induced silencing complex (Westholm and Lai, 2011; Ha and Kim, 2014; Bartel, 2018; O’Brien et al., 2018).

The mechanisms that regulate miRNA transcription, a key factor of miRNA abundance and tissue-specific expression, are not well defined, in particular for intronic miRNAs. Intronic miRNAs were first observed as frequently co-regulated with their host genes (Rodriguez et al., 2004; Seitz et al., 2004; Baskerville and Bartel, 2005; Liang et al., 2007; He et al., 2012), suggesting that their transcription depends on the promoter activity of the host gene. By contrast, recent work suggests that most intronic miRNAs are not co-regulated with their host genes and they have independent transcription start sites (Steiman-Shimony et al., 2018). Many additional studies have tried to map miRNA promoters using bioinformatics tools (Chen et al., 2019). For instance, genome-wide mapping of cardinal features of promoters, including RNA PolII or PolIII binding, enrichment in specific histone marks, transcription factor binding sites and depletion in nucleosomes, shows that 30%–35% of miRNA have independent promoters (Ozsolak et al., 2008; Monteys et al., 2010) of miRNA have independent promoters. The location of miRNA promoters was also inferred from CAGE data (Cap analysis gene of expression) (Chien et al., 2011; Marsico et al., 2013). Recently, de Rie and coworkers as part of the FANTOM5 project (Functional Annotation of Mammalian Genome) have combined CAGE and RNA-seq data to locate TSS and the 5’ end of pri-miRNAs in many tissues and cell types. They estimated that there are 1,357 promoters for humans and 804 for mouse miRNAs (de Rie et al., 2017). However, these putative regulatory regions were not functionally validated.

CRISPR (Clustered regularly interspaced short palindromic repeats)/Cas9 activation (CRISPRa) and inhibition (CRISPRi) are two techniques that take advantage of a dead Cas9 (dCas9) that is fused to either activators or repressors of transcription. CRISPRi or CRISPRa complexes are targeted to a specific genomic region using a complementary single guide RNA (sgRNA), which results in an increase (for CRISPRa) or a decrease (for CRISPRi) in the expression levels of the targeted gene (Gilbert et al., 2013; Konermann et al., 2015; Lee et al., 2016; Yeo et al., 2018). CRISPRa/i tools have been previously used to test the function of long non-coding RNAs (Liu and Lim, 2018; Zhao et al., 2021). Hence, we reasoned that CRISPRa/i might be used to map miRNA promoters and validate predictions made using bioinformatics tools. As a proof of principle, here CRISPRa or CRISPRi complexes were directed to the predicted regulatory sequences of two intronic miRNAs, *mmu-miR-335* and *hsa-miR-3662*, including intronic sequences and the promoters of their respective host genes *Mest* and *HBS1L*.

## Material and methods

### Cell culture

#### Mouse embryonic stem cells (mESCs) and mESCs-derived brain organoids

E14Tg2a mESCs were cultivated on gelatine-coated dishes and maintained pluripotent in Serum/Lif media as described (Varrault et al., 2018).

Brain organoids were generated in 96-well (U-bottom) Ultra-Low Adhesion plates (Sumitomo) by seeding 3,000 ESCs in corticogenesis medium 1: DMEM/F-12/GlutaMAX supplemented with 10% KSR, 0.1 mM of non-essential amino acids, 1 mM of sodium pyruvate, 50 U/mL penicillin/streptomycin, 0.1 mM of 2-mercaptoethanol (Sigma), 1 µM DMH1-HCl (in house synthesized, Vanderbilt University) and 240 nM IWP-2 (Tocris). On day 8 of differentiation, organoids were transferred to bacterial plates (Greiner) in corticogenesis medium 2: DMEM/F-12/GlutaMAX supplemented with N2 and B27 (without vitamin A) supplements, 500 μg/mL of BSA, 0.1 mM of non-essential amino acids, 1 mM of sodium pyruvate, 0.1 mM of 2-mercaptoethanol, and 50 U/mL penicillin/streptomycin (Le Digarcher et al., 2022).

#### Mouse embryonic fibroblasts (MEFs)

Immortalized CRISPRa (SAM) MEFs (gift from Giacomo Cavalli’s lab, unpublished) were cultivated in DMEM supplemented with 10% FBS and 50 U/mL penicillin/streptomycin. All media components were from Life Technologies unless otherwise stated. Cell lines were routinely tested for the absence of *mycoplasma* (Mycoalert, Lonza).

#### HEK-293T cells

HEK-293T cells were obtained from the American Type Culture Collection (Manassas, VA). They were grown and maintained in high-glucose DMEM supplemented with 10% FBS and 1% penicillin/streptomycin (100 U/L penicillin and 100 U/L streptomycin).

### CRISPRa and CRISPRi cell lines

#### Generation of CRISPRi (dCas9-KRAB-MeCP2) mESCs

To generate CRISPRi mESCs, E14Tg2a mouse ESCs were co-transfected with pCMV-HA-HyperpiggyBase (Yusa et al., 2011)–pB- and pB-dCas9-KRAB-MecP2 (Yeo et al., 2018) (Addgene plasmid # 110824) using a Neon transfection system (Life Technologies). Forty-8 hours post-transfection, cells were selected using Blastidicin (15 μg/mL, SIGMA). The detailed protocol to generate CRISPRi and CRISPRa cell lines was previously published (Le Digarcher et al., 2022).

#### Generation of CRISPRa (SAM) HEK-293 cells

To generate CRISPRa HEK293 cells, the pB-SAM (Addgene, #102559) and HyperpiggyBase plasmids were co-transfected using Lipofectamine 3,000 (Thermo Fisher Scientific). HEK-293 colonies stably expressing the CRISPRa system were selected with blasticidin (10 μg/mL) and validated by Western blot using an anti-Cas9 antibody.

#### CRISPRa (SAM) mESCs and SAM MEFs

SAM mESCs (Bonev et al., 2017) and SAM MEFs were a gift from Giacomo Cavalli’s lab.

### Generation and design of sgRNAs

sgRNA sequences targeting *Mest* promoters were designed using CRISPick https://portals.broadinstitute.org/gppx/crispick/public (formerly GPP sgRNA Design tool) or manually. sgRNAs that target the putative *miR-335* promoter (mm10_dna range = chr6_30740830–30741300) were designed using CHOPCHOP (Labun et al., 2019).

#### Plasmids

Pairs of oligonucleotides (Eurofins) were annealed and subcloned into either sgRNA (MS2) cloning backbone (Addgene Plasmid #61424) or Lenti sgRNA (MS2)_zeo backbone (Konermann et al., 2015) (Addgene plasmid # 61427) that were previously digested with either BbsI or BsmBI (NEB), respectively, and purified on a Chromaspin column (Clontech). All constructs were verified by Sanger sequencing (Genewiz). sgRNA sequences are listed in Supplementary Table S1.

#### Lentiviruses

Lentiviruses were prepared as described elsewhere (Lin et al., 2002). Briefly, lentiviral transfer vectors were co-transfected with the HIV packaging plasmid psPAX2 and the plasmid pMD2G (coding for the vesicular stomatitis virus envelope glycoprotein G), in HEK-293T cells by the calcium phosphate method. Supernatants were collected on day 2 post-transfection and concentrated on sucrose by ultracentrifugation at 95 528 g for 1.5 h at 4°C.

### Expression of sgRNAs in CRISPRa/i cells by lentiviral transduction or transfection

#### Generation of SAM CRISPRa ESC lines expressing sgRNAs that target *Mest* promoters

E14Tg2a ESCs stably expressing the SAM system (Bonev et al., 2017) –SAM ESCs-were transfected with Lenti sgRNA (MS2)_zeo plasmids expressing the following sgRNAs: control, *Mest* distal promoter, *Mest* proximal promoter#1, or *Mest* proximal promoter#2. ESCs were selected using Zeocin (250 μg/mL, Life Technologies) and clones were picked and expanded.

#### Generation of CRISPRi ESC lines expressing sgRNAs that target the promoters of *Mest*

CRISPRi ESCs were transduced with lentiviruses expressing the following sgRNAs: control, *Mest* distal promoter, *Mest* proximal promoter#1, or *Mest* proximal promoter#2. Seventy-2 hours post-infection, cells were selected using hygromycin (1 mg/mL, Life Technologies), and clones were picked and expanded.

#### Transient transfection of sgRNAs in SAM MEFs

80,000 MEFs stably expressing the SAM system (SAM MEFs) were transfected using Lipofectamine 2000 with 300 ng of sgRNA (MS2) plasmid expressing either one control sgRNA, one *Mest* distal promoter sgRNA (out of 3 different sgRNAs), one *Mest* proximal promoter sgRNA (out of 2 different sgRNAs), or one *miR-335*-putative promoter sgRNA (out of 3 different sgRNAs). Forty-8 hours later, RNAs were harvested.

#### Transient transfection of sgRNAs in SAM HEK293 cells

3 μg of lenti-sgRNA-(MS2)-zeo plasmid expressing either a control sgRNA, sgRNAs targetting miR-3662 or *HBS1L* promoters were transfected into CRISPRa HEK-293 cells using Lipofectamine 3,000. RNAs were collected 24 h, 48 h, and 72 h after transfection.

### RNA extraction and gene quantification

For *miR-335* and *Mest*, total RNAs were extracted using quick-RNA miniprep kits (Zymo) and quantified on a Nanodrop. RNAs were retro-transcribed with N6 primers and M-MuLV retro-transcriptase (RT). qPCR was performed using validated primers and SYBR Green Mix (Roche) in 384-well plates on a LightCycler480 device (Roche) as described in (Bouschet et al., 2017). Each gene’s expression level was normalized to the average expression levels of three housekeeping genes selected with geNorm (Vandesompele et al., 2002): *Gapdh*, *Tbp*, and *Mrpl32* for ESCs and *Gapdh*, *Tbp* and *Gusb* for MEFs (and *Gapdh* and *Tbp* when comparing ESCs to MEFs). miRNAs were retro-transcribed with gene-specific primers and multiscribe RT (Life Technologies). Their levels of expression were measured with TaqMan probes (miRNA Taqman assays # 000546 for *miR-335*-5p, and # 002185 for *miR-335*-3p). and normalized to that of U6 snoRNA (assay # 001973) (ThermoFisher). U6 was found to be stably expressed across samples (not shown).

For *miR-3662* and *HBS1L*, 1 μg of RNA was reverse transcribed using miScript II RT Kits (QIAGEN, Hilden, Germany) or Mir-X miRNA qRT-PCR TB Green Kit (Takara Bio United States, Inc.) according to the manufacturer’s protocol. Then, cDNA was used as a template for real-time PCR using a QuantStudio 3 (Applied Biosystems, Waltham, MA) with miScript SYBR Green PCR kits (QIAGEN) at 95°C for 15 min, followed by 40 cycles of 95°C for 15 s, 55°C for 30 s and 72°C for 30 s or Mir-X miRNA qRT-PCR TB Green Kit (Takara Bio United States, Inc.) 95°C for 10 s, followed by 40 cycles of 95°C for 5 s, 60°C for 20 s. For coding genes, 1 μg of RNA was reverse-transcribed using a High-Capacity cDNA Reverse Transcription Kit (Catalog number: 4,368,813). Then, cDNA was used as a template for real-time PCR using QuantStudio 3 (Applied Biosystems, Waltham, MA) with SYBR™ Green PCR Master Mix (Catalog number: 4,309,155). The relative quantities of miRNA and coding gene were determined by the comparative method (2^−ΔΔCT^) with a U6 and GAPDH respectively as a reference.

qPCR primer sequences are listed in Supplementary Table S2.

### Immunofluorescence

Immunofluorescence experiments were performed as described (Varrault et al., 2018) using antibodies directed against (species; provider; catalogue number): CAS9 (mouse; Cell signalling; #14697); NANOG (mouse; BD Pharmingen; #560259); NESTIN (mouse; Santa Cruz; sc-33677); PAX6 (mouse; Covance; PRB-278P); POU5F1 (rabbit; Cell signalling; #2840); TBR1 (rabbit, Cell signalling; #49661); TUBB3 (mouse; Covance; MMS-435P). Secondary antibodies were anti-mouse or anti-rabbit coupled to Alexa Fluor^®^ 488 or Cy3 (Jackson Immunoresearch Laboratories). Nuclei were labelled with DAPI and slides were mounted with mowiol and observed under a fluorescence microscope (ImagerZ1, Zeiss). Images of organoids were obtained by tiling and stitching, and insets were taken using the apotome mode.

### Statistical analysis

Statistical analysis was carried out using GraphPad Prism Version 8 (GraphPad Software, San Diego, United States) and the test names are indicated in the figure legends.

## Results

### Development of a pipeline to test the activity of putative miRNA promoters with CRISPRa and CRISPRi

**FIGURE 1:**
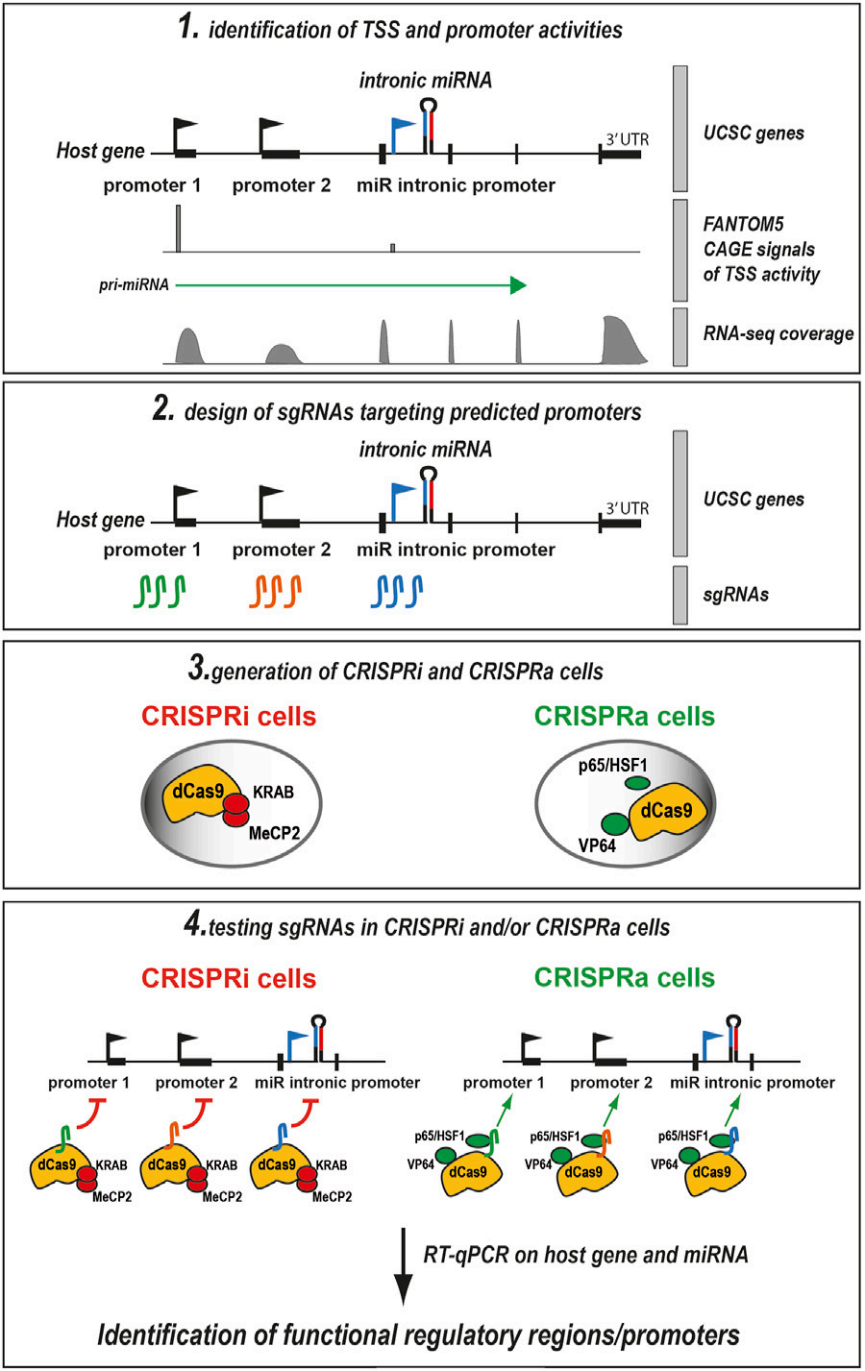
Flowchart of the four-step strategy to test the functional role of genomic regions in miRNA expression. Step 1: The putative promoters are inferred from CAGE data (here from the FANTOM5 consortium, which also predicts pri-miRNA transcription start), and/or RNA-seq data. In the depicted example, the host gene has two predicted promoters and the intronic miRNA has one promoter. CAGE reads map to the host gene promoter and to a lesser extent to the intronic miR promoter, indicating that these two promoters are active (preferentially the host gene promoter 1 over the miRNA intronic promoter). By contrast, the host gene promoter 2 is inactive (no CAGE reads). CAGE data can be corroborated with RNA-seq data (here, they confirm that the host gene promoter 1 is active). Step 2: two to three sgRNAs are designed to target the three putative promoter regions (green sgRNAs for host gene promoter 1, orange sgRNAs for host gene promoter 2, and blue sgRNAs for the miRNA intronic promoter). Step 3. Production of CRISPRi and CRISPRa cell types of interest that express dCas9 fused to the repressors of transcription KRAB and MeCP2 (CRISPRi) or the transactivators p65, HSF1, and VP64 (CRISPRa). Step 4: sgRNAs are expressed in CRISPRi and/or CRISPRa cells and the levels of the host gene and miRNA are quantified by RT-qPCR. If the level of the miRNA is affected by certain sgRNAs in CRISPRa/i experiments, this suggests that the targeted genomic region has a functional impact on miRNA expression. Adapted with permission from (Le Digarcher et al., 2022). TSS: transcription start site.

To test the function of regulatory regions on miRNA expression, we designed an experimental pipeline in four steps (Figure 1). The first step consists in locating active promoters using CAGE and/or RNA-seq data, which are either homemade or publicly available from consortiums, including the FANTOM5 consortium (de Rie et al., 2017). In the example depicted in Figure 1, the host gene has two promoters and the intronic miRNA has one promoter. FANTOM5 CAGE data (de Rie et al., 2017) peaks colocalize with promoter 1 (host gene) and the intronic miRNA promoter, showing that these promoters are active in this cell type. RNA-seq data confirms that promoter 1 of the host gene is active. The FANTOM5 further predicts that the pri-miRNA originates at promoter 1 of the host gene. Then, in step 2, two to three sgRNAs are designed to target these genomic regions. In parallel (step 3), CRISPRi and CRISPRa modules are stably introduced in the cell type of interest. Here, for CRISPRi we have used a dCas9 fused to the repressors of transcription KRAB and MeCP2 (Figure 1). dCas9-KRAB-MeCP2 was previously shown to be efficient for repressing a vast panel of genes in HEK-293T cells (Yeo et al., 2018). The CRISPRa system was composed of dCas9 and three transactivators: VP64, p65 and HSF1 (Konermann et al., 2015). This CRISPRa tool is also known as SAM (for Synergistic Activation Mediator (Konermann et al., 2015)) and is considered one of the most efficient CRISPRa systems. Finally, in step 4, sgRNAs are expressed in CRISPRi or CRISPRa cells and the levels of the host gene and the miRNA are quantified by RT-qPCR. If the level of the miRNA is affected by certain sgRNAs, this suggests that the targeted genomic region has a functional role in the expression of the miRNA.

### CRISPRi-mediated repression of Mest promoter suppresses the expression of hosted miR-335 in embryonic stem cells

**FIGURE 2:**
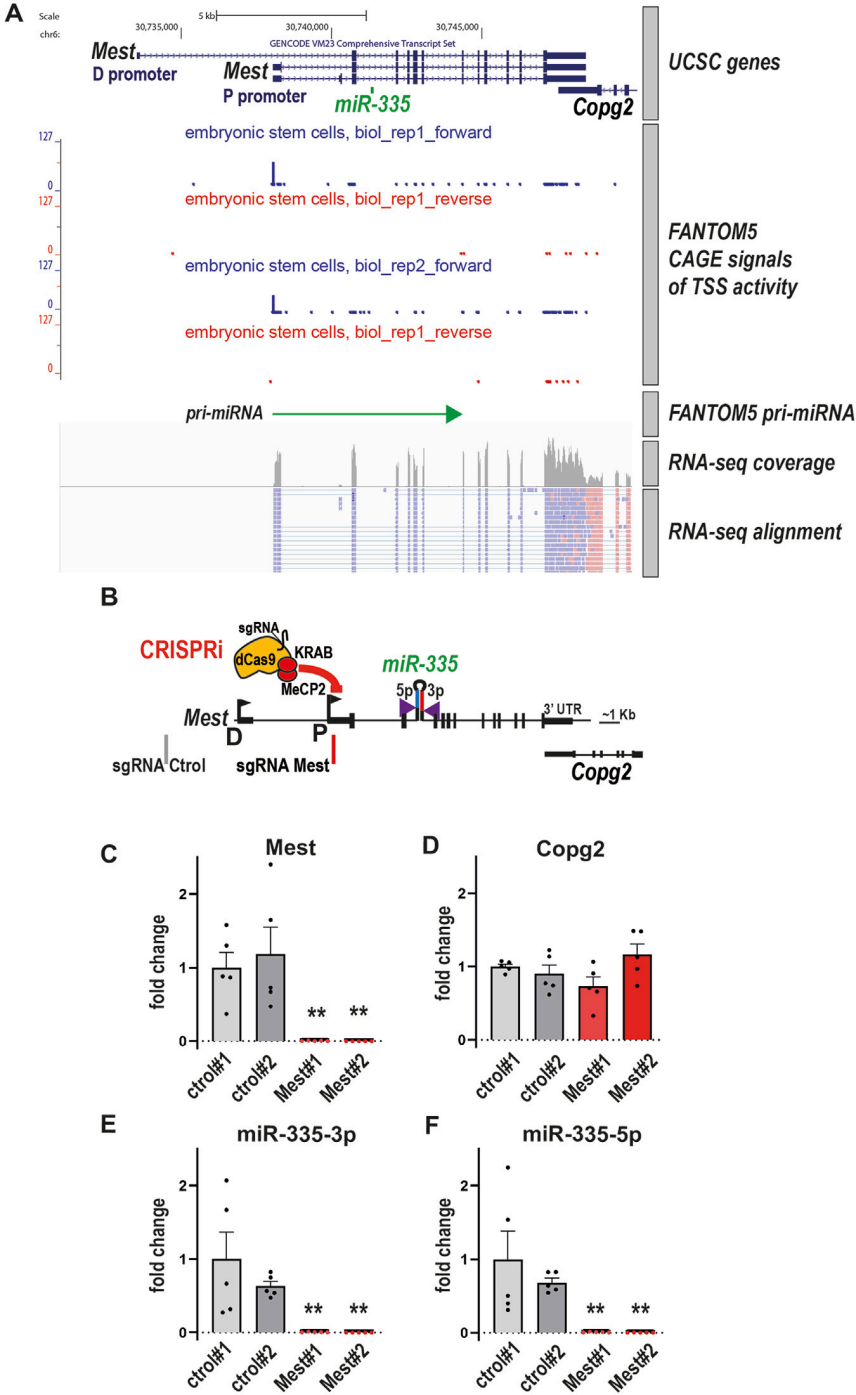
CRISPRi-mediated repression of Mest promoter suppresses the expression of hosted miR-335 in embryonic stem cells **(A)** Transcription of *Mest* and pri-miRNA-335 originate from the proximal promoter of *Mest* (P) in mouse embryonic stem cells. From top to bottom panels: genome organisation of the *Mest* gene with *Mest* distal (D) and proximal (P) promoters and *miR-335* located in an intron of *Mest*. Chromosomal coordinates and gene annotation are from the RefSeq mm10 build. CAGE signals of TSS activity in ESCs–in duplicate, ES-46C embryonic stem cells, neuronal differentiation, day00, biol_rep1 and 2 - and the pri-miRNA TSS are mapped at the *Mest* P promoter (data are from the FANTOM5 database (de Rie et al., 2017; Lizio et al., 2019)). Integrative Genomics Viewer capture shows coverage plot and alignment of RNA-seq reads in mESCs (GSE75486 (Bouschet et al., 2017)). Reads for *Mest* (blue) are transcribed from the + strand, while reads from *Copg2* (pink) are transcribed from the - strand. *Mest* transcripts originate from the P promoter and + strand, corroborating CAGE data. The *Copg2* gene is not entirely shown. D: *Mest* distal promoter; P: *Mest* proximal promoter. **(B)** Schematic of mouse *Mest* gene with the CRISPRi module (dCas9-KRAB-MeCP2) targeting the proximal promoter P of *Mest*. **(C, D)** Repression of the *Mest* promoter downregulates *Mest* **(C)** but does not affect the expression of neighbouring *Copg2* **(D)**. RNAs were quantified in two CRISPRi ESC clones expressing the control sgRNA (grey) and two CRISPRi ESC clones expressing *Mest* sgRNA (red). Data are mean ± sem of five independent experiments and expressed as fold change over control clone #1. **:*p* < 0.01 (Mann-Whitney test). **(E, F)** Repression of the *Mest* promoter downregulates *miR-335*-3p and *miR-335*-5p levels. Data are mean ± sem of five independent experiments and expressed as fold change over control clone #1. **:*p* < 0.01 (Mann-Whitney test).

We first applied our pipeline to *miR-335. miR-335* is located in an intron of the protein-coding gene *Mest* (Figures 2A, B). *miR-335* is transcribed from the same DNA strand as its host gene, a common feature of intronic miRNAs (Hinske et al., 2014). *Mest* and *miR-335* are highly conserved during evolution and frequently co-regulated (Liang et al., 2007; Ronchetti et al., 2008; Tomé et al., 2011; Yang et al., 2014; Hiramuki et al., 2015). This suggests that *Mest* and *miR-335* share common regulatory sequences. *Mest* has one distal promoter (D) and one proximal promoter (P) (Figure 2A). In addition, the PROmiRNA and FANTOM5 databases (Marsico et al., 2013; de Rie et al., 2017) suggest that the promoter of *miR-335* is the *Mest* P promoter. In mouse embryonic stem cells (mESCs), RNA-seq (Bouschet et al., 2017) and CAGE (de Rie et al., 2017) data show that *Mest* transcripts originate predominantly from the P promoter, close to the predicted TSS of the pri-miRNA (Figure 2A). (Capitano et al., 2017; Ji et al., 2017; Medley et al., 2021)

We reasoned that if *miR-335* expression depends on the activity of either *Mest* promoters, then repressing transcription at *Mest* promoters with CRISPRi should decrease *miR-335* transcripts in mESCs. Using Hyper-piggyBac transposase (Yusa et al., 2011), we first generated a CRISPRi mESC line that stably expresses dCas9 fused to the repressors of transcription KRAB and MeCP2. CRISPRi mESCs (characterized in Supplementary Figure S1) were transduced with lentiviruses that express either a control sgRNA (no match in the mouse genome) or a sgRNA targeting either the D or the P promoters of *Mest* (Supplementary Figure S2A). sgRNAs targeting *Mest* promoter P downregulated *Mest* while targeting distal promoter D had no obvious effect (Supplementary Figure S2B). Thus, as expected, CRISPRi was efficient only when targeting the active *Mest* promoter. Levels of the neighbouring gene *Copg2* were unaffected (Supplementary Figure S2C).

We then selected two CRISPRi mESC clones expressing the control sgRNA and two clones expressing the sgRNA *Mest* P2 for further analyses (Figure 2B). There was a >100-fold downregulation of *Mest* in CRISPRi *Mest* clones compared to CRISPRi control clones (Figure 2C). By contrast, *Copg2* expression was unaffected (Figure 2D). To determine whether the expression of *miR-335* depends on *Mest*, we next quantified *miR-335* in CRISPRi mESCs stably expressing either the control sgRNA or the sgRNA targeting the promoter of *Mest*. *miR-335* is known to generate two mature products, miR-335-5p, considered as the main product of the *miR-335* biogenesis pathway and miR-335-3p (or miR-335*, known as the passenger miRNA) (Kozomara et al., 2019; Medley et al., 2021). miR-335-3p was previously reported to be expressed in mESCs but the expression of miR-335-5p was not assessed in this study (Kingston and Bartel, 2019). Using gene-specific RT followed by qPCR with Taqman probes (see *Methods*) we detected both miR-335-3p and miR-335-5p in control ESCs (Figures 2E, F). In CRISPRi *Mest* clones, *miR-335*-3p and *miR-335*-5p levels were reduced to less than 1% of levels measured in CRISPRi control clones (Figures 2E, F), a massive downregulation that paralleled that of *Mest* (Figure 2C). Thus, the transcriptional activity of *Mest* proximal promoter is required for the expression of intronic *miR-335* in mESCs.

### Mest promoter activity is required for miR-335 expression in brain organoids

**FIGURE 3:**
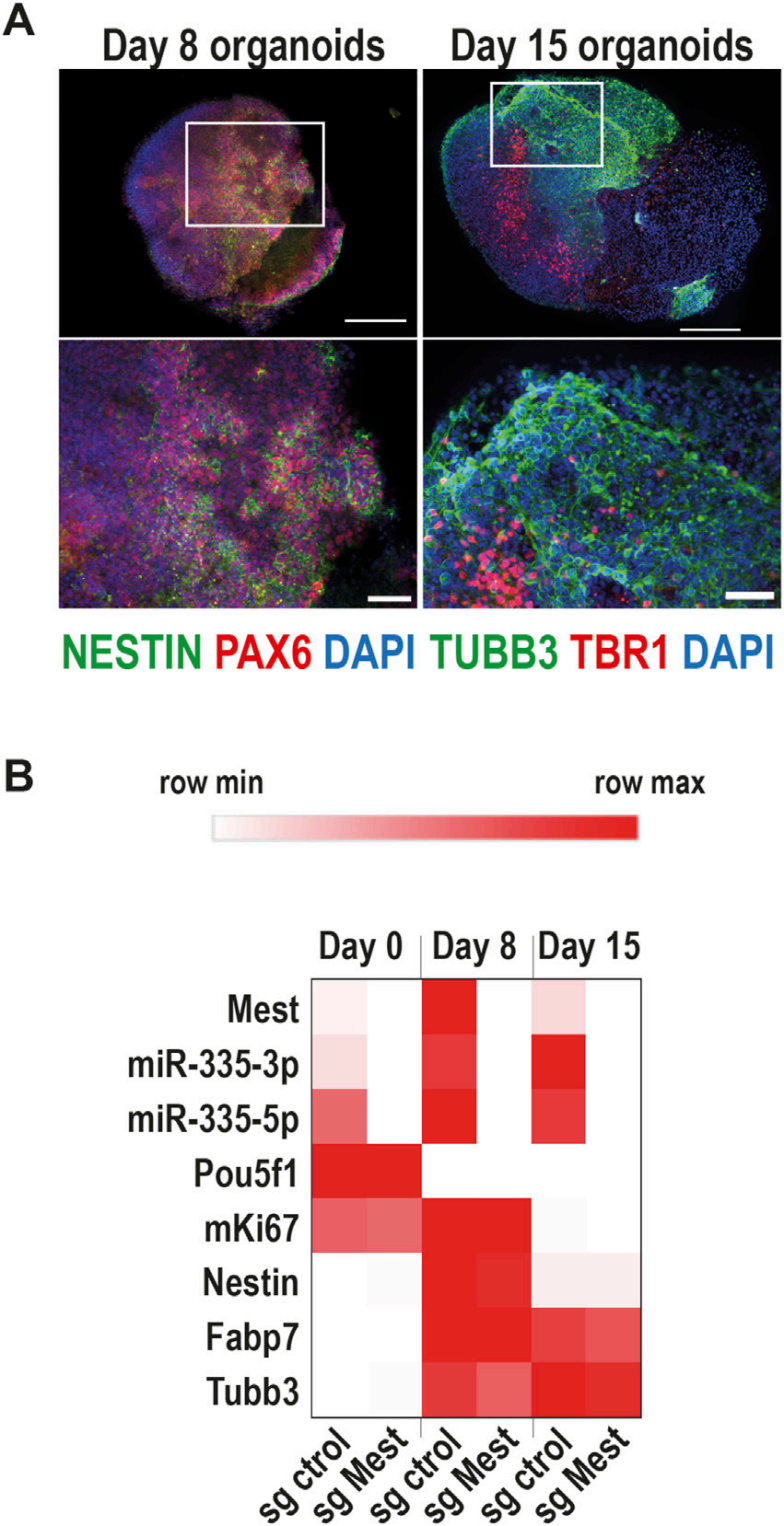
Mest promoter activity is required for miR-335 expression in brain organoids **(A)** Immunofluorescence staining on brain organoids derived from mESCs using antibodies for brain primordium markers NESTIN/PAX6 (middle panels) and TUBB3/TBR1 (right panels) after eight and 15 days of differentiation. The top panels show entire organoids and the bottom panels are zoom-in insets. Scale bars: 200 µm for organoids (top panels) and 50 µm for insets (bottom panels). **(B)** Time course of expression of *Mest* and *miR-335* mature products during the development of brain organoids from CRISPRi ESCs stably expressing either control sgRNA or *Mest* sgRNA. The heatmap shows the mean of four independent experiments performed on two CRISPRa sgRNA control and two CRISPRa sgRNA ESC clones. Heatmap was built using Morpheus. https://software.broadinstitute.org/morpheus/.

Because *Mest* and *miR-335* are co-expressed in the nervous system, where they play functional roles (Capitano et al., 2017; Ji et al., 2017), we next determined whether we could downregulate both *Mest* and *miR-335* via CRISPRi targeting of the Mest promoter in brain cells. Brain organoids were generated from CRISPRi mESCs in ultra-low adhesion plates according to a ground-breaking protocol (Eiraku et al, 2008), which we slightly modified (Le Digarcher et al., 2022). As expected, CRISPRi mESCs-derived organoids contained neural progenitors of dorsal identity (NESTIN + PAX6+ cells) after 8 days of differentiation, and neurons (TUBB3+ cells), including neurons that expressed the cortical marker TBR1 after 15 days of differentiation (Figure 3A). The neural identity of organoids was further confirmed by RT-qPCR using primers for Nestin (neural progenitors), Fabp7 (radial glia) and Tubb3 (neurons) (Figure 3B).

*Mest* and *miR-335* transcripts were co-upregulated during the generation of brain organoids from CRISPRi mESCs expressing the control sgRNA (Figure 3B). By contrast, *Mest* RNA was barely detectable in CRISPRi organoids expressing the *Mest* sgRNA (Figure 3B), showing that the *Mest* promoter remains repressed in differentiated cells. Importantly, *miR-335* mature products were also barely detectable in these organoids (Figure 3B). Thus, the activity of the *Mest* promoter is required for *miR-335* expression in both undifferentiated mESCs and their neural progeny.

### CRISPRa-induced upregulation of *Mest* induces the expression of *miR-335* in ESCs

**FIGURE 4:**
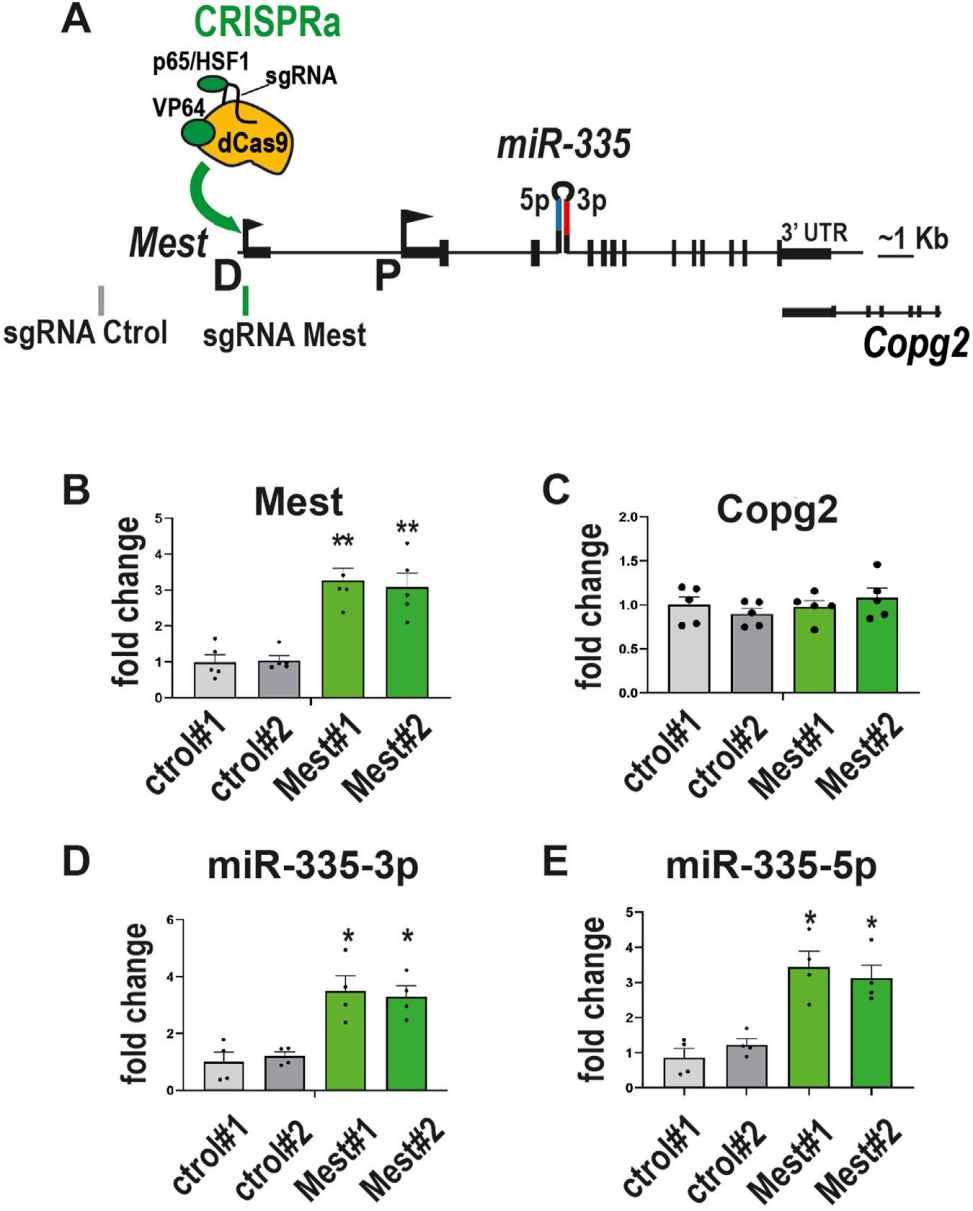
CRISPRa-induced upregulation of Mest induces the expression of miR-335 in ESCs **(A)** Schematic of mouse *Mest* gene structure with the CRISPRa SAM - synergistic activation mediator-module targeting the distal promoter D of *Mest*. **(B, C)** Transactivation of the *Mest* promoter upregulates *Mest* **(B)** but does not affect neighbouring *Copg2* expression **(C)**. Data are mean ± sem of five independent experiments performed on two CRISPRa sgRNA control (grey) and two CRISPRa sgRNA *Mest* clones (green) and expressed as fold change over control clone#1.**:*p* < 0.01 (Mann-Whitney test). **(D, E)** Transactivation of the *Mest* promoter increases *miR-335*-3p **(D)** and *miR-335*-5p **(E)** levels. Data are mean ± sem of four independent experiments and expressed as fold change over control clone#1. *:*p* < 0.05 (Mann-Whitney test).

Next, we tested whether transactivating the *Mest* promoter using CRISPRa is sufficient to increase *miR-335* levels. To this aim, mESCs stably expressing the CRISPRa system SAM (Bonev et al., 2017) were transduced with lentiviruses expressing either a control sgRNA or a sgRNA targeting *Mest* (D) or (P) promoters (Supplementary Figure S3A) –and as described for CRISPRi-. Transactivating the distal promoter of *Mest* efficiently increased *Mest* transcripts (Supplementary Figure S3B). By contrast, transactivating *Mest* proximal promoter with sgRNAs P1 and P2 had no major effect on the *Mest* transcript level (Supplementary Figure S3B), likely because this promoter is already active in ESCs (Figure 2A). The level of *Copg2* was not altered by any of the three *Mest* sgRNAs (Supplementary Figure S3C). We selected for further analysis two CRISPRa control and two CRISPRa *Mest* clones (expressing the D sgRNA, Figure 4A). On average, there was a 3.2-fold increase in *Mest* transcript in CRISPRa *Mest* clones compared to control clones (Figure 4B). *Copg2* expression was unaffected (Figure 4C). Strikingly, the levels of both *miR-335*-3p and *miR-335*-5p also increased by a ∼3-fold factor (Figures 4D, E). Thus, activating the distal promoter of *Mest* with CRISPRa/SAM is sufficient to increase the levels of hosted *miR-335*.

### CRISPRa on the intronic promoter of *miR-335* does not affect *miR-335* levels in MEFs

A previous study based on luciferase assays performed in HEK-293T cells suggests that the sequence upstream of *miR-335* (situated in a *Mest* intron and named pro2) has some promoter activity (Zhu et al., 2014). Thus, we next tested whether we could upregulate *miR-335* by directing the SAM complex to this genomic region.

**FIGURE 5:**
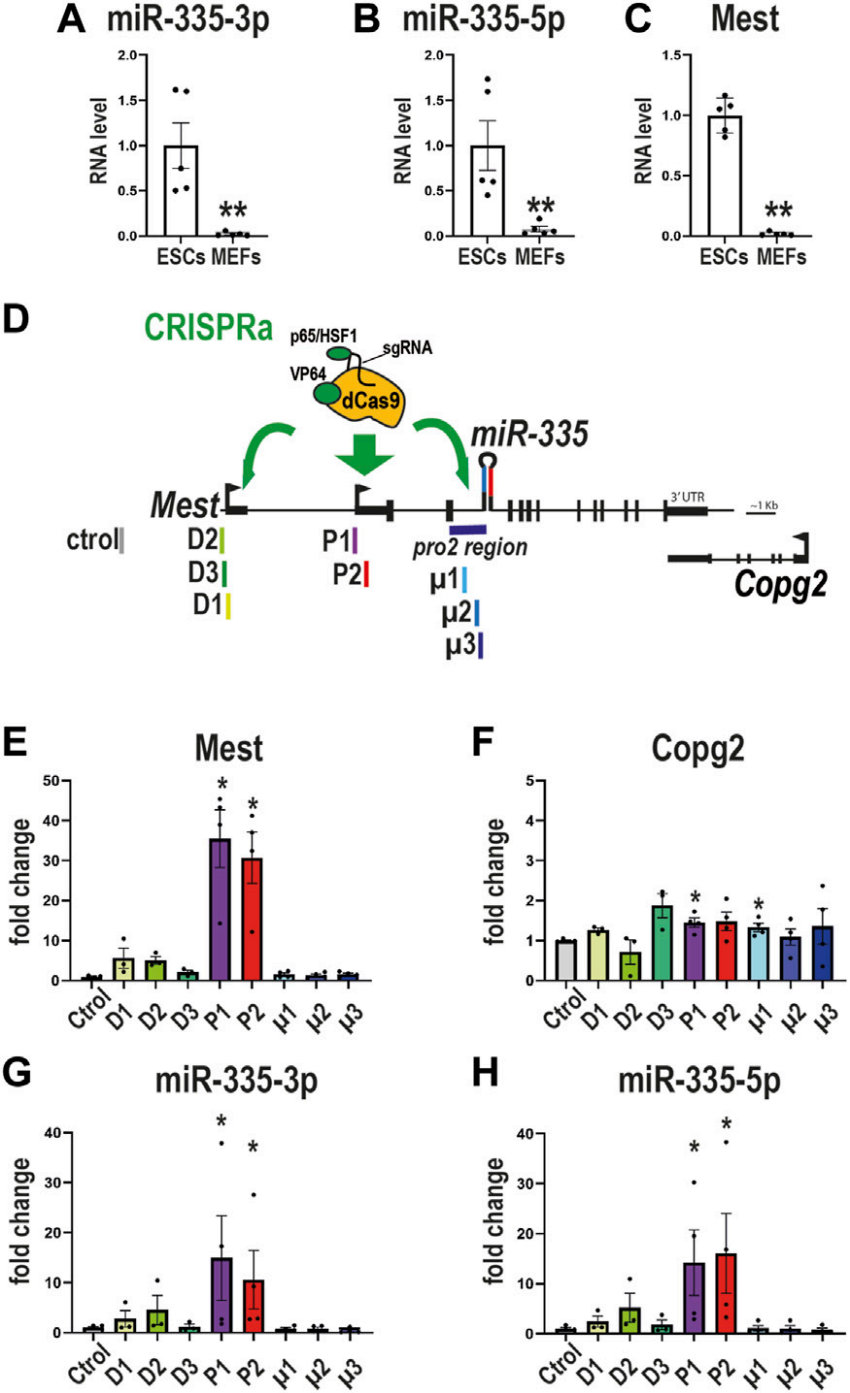
CRISPRa on the intronic promoter of miR-335 does not affect miR-335 levels in MEFs **(A–C)** Lower expression of *miR-335* and *Mest* in SAM MEFs compared to SAM ESCs. Data are mean ± sem of qPCR experiments performed on five MEF and five ESC samples and normalized to the average value obtained on ESCs.**:*p* < 0.01 (Mann-Whitney test). **(D)** Structure of mouse *Mest* gene with SAM targeting either the distal promoter of *Mest* D (D1-D2 sgRNAs), the proximal promoter of *Mest* P (P1-P3 sgRNAs), or the putative promoter of *miR-335* (µ1-µ3 sgRNAs, which target the pro2 region). **(E–H)** Levels of expression of *Mest* **(E)**, *Copg2* **(F)**, *miR-335-3p* **(G)**, and *miR-335-5p* **(H)** were measured after transactivation of either *Mest* D or P promoters or *miR-335* putative promoter. SAM MEFs were transfected with plasmids expressing sgRNAs targeting *Mest* D (sgRNAs D1, D2, and D3), *Mest* P (P1 and P2), or the putative promoter of *miR-335* (µ1, µ2, and µ3). Data are mean ± sem of three to four independent experiments and expressed as fold change over sgRNA control taken as 1. *: *p* < 0.05 in Mann-Whitney test (comparison with sgRNA control values). None of the sgRNAs that direct the CRISPRa/SAM machinery towards the *miR-335* putative promoter (µ1, µ2, and µ3) altered *miR-335* levels.

Because SAM efficiency correlates with the baseline expression levels of the targeted gene–the fold of upregulation is inversely correlated with basal transcript level- (Konermann et al., 2015), we reasoned that to maximize the chance to increase *miR-335*, these SAM experiments should be performed in cells with lower baseline levels of *miR-335* than mESCs. MEFs (mouse embryonic fibroblasts) were reported to express miR-335-5p (Kingston and Bartel, 2019). We observed that MEFs expressed both miR-335-3p and miR-335-5p (Figure 5A). *miR-335*-3p and *miR-335*-5p levels were respectively 13 and 47 times lower in MEFs compared to mESCs (Figures 5A, B). *Mest* expression was also ∼60 times less expressed in MEFs than in mESCs (Figure 5C), adding further support for the coregulation of *Mest* and *miR-335*. Thus, MEFs have low baseline levels of endogenous *miR-335* and *Mest* and they seem an appropriate model to perform CRISPRa experiments with maximised chances to observe an effect on *miR-335* levels.

To test the putative *miR-335* promoter (pro2 (Zhu et al., 2014), which has 96.8% sequence homology between mouse and Humans), we designed three sgRNAs (µ1, µ2, and µ3) and compared their efficiency in upregulating *miR-335* to that of sgRNAs that target *Mest* promoters (Figure 5D). We observed that sgRNAs P1 and P2 (which target *Mest* P promoter) strongly upregulated *Mest* (Figure 5E) and had no major effects on Copg2 expression (Figure 5F). sgRNAs P1 and P2 also strongly upregulated *miR-335* mature products in SAM MEFs (Figures 5G, H). The upregulation of *Mest* was much higher in MEFs than in mESCs, as expected from their relative *Mest* baseline levels (Figure 5C). By contrast, the three sgRNAs that target the putative promoter of *miR-335* (sgRNAs µ1, µ2, and µ3) did not affect *miR-335*-3p nor *miR-335*-5p levels (Figures 5G, H). Thus, this genomic sequence likely does not regulate *miR-335* expression in MEFs.

### Transactivation of the promoters of *miR-3662* or host gene *HBSIL* increases *miR-3662* levels

**FIGURE 6:**
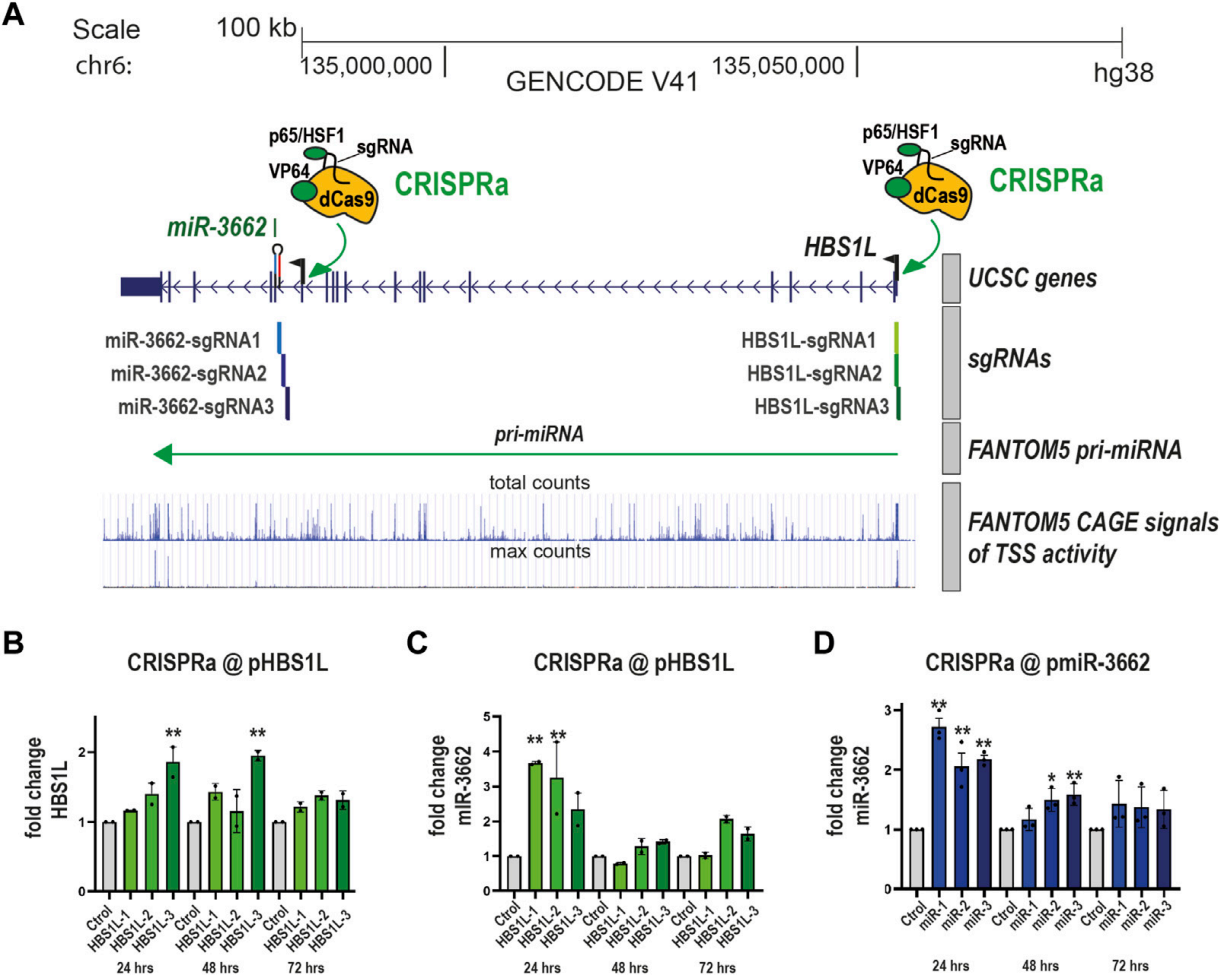
Transactivation of the promoters of miR-3662 or host gene HBSIL increases miR-3662 levels **(A)** Structure of the human *HBS1L* gene (modified screen capture from the UCSC genome browser) with the SAM complex targeting either the promoter of *HBS1L* (sgRNAs HBS1L 1–3) or the promoter of *miR-3662* (sgRNAs *miR-3662 1*–*3*), which is located in an intron of *HBS1L*. According to FANTOM5 data, transcription of *HBS1L and* pri-miRNA-3662 both originate from the promoter of *HBS1L*. Chromosomal coordinates and gene annotation are from the RefSeq hg38. CAGE signals (max and total read counts on the reverse strand) are from the FANTOM5 database. **(B–D)** SAM HEK-293T cells were transiently transfected with the three sgRNAs targeting the promoter of *HBS1L* **(B, C)** or the 3 sgRNAs targeting the promoter of *miR-3662 (miR1-3)* **(D)**. The expression of *HBS1L* **(B)** and *miR-3662* **(C, D)** was quantified by RT-qPCR after 24, 48, or 72 h of transfection. Tukey’s multiple comparison tests were performed. *: *p* < 0.05 and **: *p* < 0.01 (comparison with sgRNA control value at the same time after transfection).

Next, we sought to extend our approach to another intronic microRNA, *hsa-miR-3662* (hereafter: *miR-3662*)*. miR-3662* is a human-specific miR that plays bivalent roles in cancer, acting either as a tumour suppressor or an oncogene in different cellular contexts (Powrózek et al., 2015; Maharry et al., 2016; Powrózek et al., 2017). We recently reported that *MIR-3662*, located in an intron of the *HBSL1* gene, is tunable by CRISPRa/i targetting of an intronic sequence in *HBSL1* (Yi et al., 2022). The FANTOM5 database (de Rie et al., 2017) predicts from CAGE data that *MIR-3662* uses the promoter of its host gene *HBSL1*. Thus, we next investigated whether the expression level of *miR-3662* is also tunable by activating the promoter of *HBS1L*. We first generated a HEK-293 cell line that stably expresses the CRISPRa SAM system using PB-SAM (Supplementary Figure S4). Next, SAM HEK-293 cells were transfected with plasmids expressing sgRNAs that target the *HBS1L* promoter (3 sgRNAs, called HBS1L H1-3), a control sgRNA, or 3 sgRNAs, called miR-3662-sgRNA1-3, that target the *miR-3662* promoter (in an intron of *HBS1L,* Figure 6A) and which we previously found efficient with the CRISPRa system VPR (Yi et al., 2022). The expression levels of *HBS1L* and *miR-3662* were monitored by qPCR at 24 h, 48 h and 72 h after transfection. As expected, *HBS1L* was upregulated when targeting its promoter with CRISPRa (Figure 6B). *miR-3662* was also transiently upregulated by targeting its host gene *HBS1L* (Figure 6C). In parallel, *miR-3662* was upregulated by targeting its promoter with SAM and sgRNAs-miR-3662 (Figure 6D). Thus, *miR-3662* can be activated by targeting both its own promoter and its host gene promoter in HEK-293T cells.

## Discussion

Here we show that CRISPRa and CRISPRi, where dCas9 is fused to transcriptional activators or repressors, respectively, are powerful tools to test genomic regions predicted to be microRNA promoters in databases such as those released by the FANTOM5 consortium (de Rie et al., 2017).

Others have previously used CRISPR-based approaches to manipulate miRNA levels and/or map their promoters. As early as 2014, Zhao and colleagues observed that dCas9 targeted to the upstream sequence of the miR cluster *miR17-92* leads to the repression of *miR-19a*, *miR-20a* and *miR-92* (Zhao et al., 2014). In these experiments, dCas9 was not fused to a transcriptional repressor. Thus, Zhao and colleagues hypothesized that dCas9 sterically blocked RNA PolII (hence they have used the term CRISPR interference and not CRISPR inhibition) (Zhao et al., 2014). More recently, Drobna-Śledzińska and colleagues have developed a complete pipeline from microRNA TSS prediction to sgRNA design and CRISPR inhibition. TSS prediction was inferred from four databases (miR start, DIANA-miRGen, CRISPR tool and FANTOM 5) (Drobna-Śledzińska et al., 2022). Here, as described by Drobna-Śledzińska and colleagues, we have used FANTOM 5 as a prediction tool for promoter activity and CRISPRi. We have further completed the prediction of FANTOM5 with RNA-seq experiments. Prediction tools, including FANTOM5, predict active promoters. Hence, these tools are useful to design sgRNAs for CRISPRi experiments.

In addition to CRISPRi, we also performed CRISPRa experiments. We observed that CRISPRa did not upregulate Mest RNA when the SAM complex targeted the *Mest* proximal promoter (predicted to be active in mESCs by FANTOM 5). By contrast, CRISPRa was effective when targeting the inactive *Mest* promoter (hence absent from the FANTOM5 database). Collectively, this suggests that sgRNAs for CRISPRi should target the active promoter (predicted in databases such as the FANTOM5 database), while for CRISPRa it is worth targeting all of the known promoters, especially those that are inactive.

This also likely depends on the basal level of expression and cell type. Indeed, our data obtained in MEFs also revealed that transactivating the (P) promoter resulted in a strong increase in *Mest* and *miR-335* while transactivating the (D) promoter had moderate effects. This contrasts with results obtained in ESCs where the most potent sgRNAs were those targeting the (D) promoter (Figure 4 and Supplementary Figure S3). Thus, taken together, these data suggest that transcriptional activation of one or the other *Mest* promoter is sufficient to increase the levels of intronic *miR-335*. This also suggests that this CRISPR-based approach is effective to locate miR regulatory sequences in a cell-type-specific manner. Finally, it also supports the existence of primary transcripts, originating either at (D) or (P) promoters that contain both *Mest* and *miR-335* precursors.

From CAGE data, the FANTOM5 consortium predicts that the promoters of *mmu-miR-335* and *hsa-miR-3662* are the promoters of their host genes (*Mest* and *HBS1L*, respectively), which we verified experimentally here.

Indeed, the activity of the *Mest* promoter was both necessary (as shown by CRISPRi) and sufficient (as shown by CRISPRa) to drive *miR-335* expression. In addition, *miR-335* was upregulated by activating different *Mest* promoters: the proximal promoter (located in the imprinted centre control region of this locus (Lefebvre et al., 1998)) in mESCs and the distal promoter in fibroblasts. This shows that CRISPR can reveal the regulatory regions involved in miRNA expression in specific cell types.

We also observed that in all our experimental setups (CRISPRa or CRISPRi, ESCs or MEFS, and brain organoids), the levels of *miR-335* parallel those of *Mest*. *Mest* and *miR-335* gene products might therefore converge on a common pathway. In this context, both *Mest* and *miR-335* influence neuronal migration, as shown using shRNA for downregulating *Mest* and a miR-inhibitor for *miR-335* (Ji et al., 2017). Using CRISPRa/i on the promoter of *Mest* should allow for studying the function of coregulated *Mest* and *miR-335* in one go. We did not observe major differences in the expression of the neural markers Nestin (neural progenitors), Fabp7 (radial glia) and Tubb3 (neurons) between CRISPRi Mest and CRISPRi control organoids. This suggests that neural differentiation was grossly normal (as estimated by these three typical markers) at these developmental time points when both Mest and miR-335 are strongly downregulated. A more comprehensive exploration is required to determine whether other neurodevelopmental genes are affected. In addition, it is also possible that the residual ∼1% of Mest and miR-335 expression are sufficient for normal neurodevelopment *in vitro*. On the other hand, in other tissues and cell types, *Mest* and *miR-335* might perform independent functions as shown in the muscle. Indeed, *Mest* KO animals but *miR-335* KO animals display a muscular defect (Hiramuki et al., 2015).

We failed to upregulate *miR-335* by CRISPRa-mediated targeting of its predicted promoter located in a *Mest* intron (Zhu et al., 2014). Collectively, this suggests that the expression of *miR-335* only depends on the activity of the promoter of *Mest*. We cannot rule out that *miR-335* has an independent promoter located in another region or is active in another cell type. In this context, the prediction of *miR-335* promoter location made by the PROmiRNA database (Marsico et al., 2013) suggests that there could be several *miR-335* promoters depending on the tissue, the most probable being *Mest* (D) and (P) promoters - what we confirmed experimentally here-, and less probably, a third region situated in another intron of *Mest*. It is also possible that additional regulatory sequences, including enhancers situated at long distances, influence the expression of *miR-335*. In this context, it will be interesting to screen the *Mest* locus along several Mbs with a wider range of sgRNAs, not only with the CRISPRa and CRISPRi molecular platforms as done here but also with dCas9 fused to enhancer regulators (Li et al., 2020).

The positive co-regulation of intronic microRNA and host gene strongly predicts that the microRNA’s transcription depends on its host gene’s promoter (Rodriguez et al., 2004; Seitz et al., 2004; Baskerville and Bartel, 2005; Liang et al., 2007; He et al., 2012). Previous works propose that evolutionarily conserved intronic miRNAs, such as *miR-335*, are more frequently co-expressed with their host genes than recently appeared intronic miRNAs (He et al., 2012; Steiman-Shimony et al., 2018). This suggests that the transcription of conserved intronic miRNAs depends on the host promoter while recently appeared intronic miRNAs tend to have independent promoters, which we confirmed here for the human-specific *miR-3662. miR-3662* is a recently appeared intronic miRNA and was CRISPR activable by targeting both its host gene *HBS1L* promoter and an independent promoter located in an intron of *HBS1L*. Although we previously demonstrated that targeting dCas9-KRAB to the intronic promoter of miR-3662 resulted in its downregulation (Yi et al., 2022), in the present study, we were unable to confirm our observation with CRISPRi when targeting the promoter of *HBS1L* due to the failure to establish a stably expressing CRISPR dCas9-KRAB-MeCP2 HEK-293 cell line.

Here, we have used the SAM CRISPRa system, which is composed of dCas9 linked to three transactivator domains: VP64, p65 and HSF1 (Konermann et al., 2015). The CRISPRi consisted of dCas9 fused to KRAB and MeCP2. In recent years, there has been a tremendous development of the CRISPRa/i toolkit. The current view is that activators or repressors act in synergy and that duo or trio of activators or repressors works better than alone (Konermann et al., 2015; Kampmann, 2018; Yeo et al., 2018). We have not compared the activating or repressing efficiencies of different CRISPRa and CRISPRi systems on miRNA levels. However, our approach is likely feasible using other CRISPRa/i tools. As an example, we have recently reported that *miR-3662* levels can be modified using dCAS9 fused to the VPR (CRISPRa) or KRAB (CRISPRi) (Yi et al., 2022). Drobna-Śledzińska and colleagues have also repressed several miRNAs using dCas9 fused to KRAB (Drobna-Śledzińska et al., 2022).

Nevertheless, our approach suffers from some limitations:

1/It requires that the promoter activities are defined in the cell type of interest. In addition, as mentioned above, alternative regulatory regions other than promoters are also possible. Thus, a strategy would be to screen several Mbases along the host gene/hosted miRNA locus to find regulatory regions in an unbiased manner. In this context, CRISPRi was previously used to map the regulatory elements (including distant elements) involved in MYC and GATA1 expression (Fulco et al, 2016).

2/Our approach depends on the basal level of expression of the targeted miRNA. When the miRNA is expressed at low levels, expressing the CRISPRa module and the sgRNA in a few cells is likely sufficient to exceed the low level and obtain an upregulation. By contrast, for highly expressed miRNAs, the CRISPRa complex and sgRNA should be expressed in a maximum of cells to eventually get an upregulation effect.

For CRISPRi, to obtain a good downregulation, the CRISPRi module should be expressed in all cells. To illustrate this, we obtained a much better downregulation effect on *Mest/miR-335* on clones (Figure 2), where all cells are supposed to express the CRISPRi module and the sgRNA, than on the polyclonal population (Supplementary Figure S2) which is a mix of cells with efficient and inefficient CRISPRi. Thus, collectively, our strategy is easily amenable to models that are easy to transfect (such as cell lines) but less applicable to complex tissues, which in essence are difficult to transfect.

However, this approach is compatible with organism models such as C.elegans and zebrafish, where the CRISPRa/i module and guide RNAs can be injected into one-cell stage embryos (Long et al., 2015).

3/sgRNAs can have off-target effects. Here, we focused our study on the targeted locus and we can not rule out that our sgRNAs have unintended targets. Reciprocally, for sgRNAs that did not work, such as those targeting the intronic miR-335 promoter (sgRNAs µ1-µ3), it will be worth confirming that these sgRNAs bind to their intended DNA targets, using chromatin immunoprecipitation with anti-Cas9 antibody for example.

To conclude, and despite these limitations, CRISPRa/i experiments on *miR-335* and *miR-3662* in mouse and human cells confirm the prediction that transcription of these two intronic miRNAs depends on the promoter of their host genes Whether this applies to other intronic miRNAs needs to be demonstrated.

We also observed that CRISPRa can reveal cell type-specific promoters (at least for *miR-335*). This CRISPR-based approach could be used to test the function of regulatory sequences (including microRNA promoters and enhancers) with the high genomic precision of sgRNAs on a genome-wide scale in different cell types.

## Data Availability

The raw data supporting the conclusion of this article will be made available by the authors, without undue reservation.

## References

[B1] Bartel D. P. (2018). Metazoan MicroRNAs. Cell 173, 20–51. 10.1016/j.cell.2018.03.006 29570994 PMC6091663

[B2] Baskerville S. Bartel D. P. (2005). Microarray profiling of microRNAs reveals frequent coexpression with neighboring miRNAs and host genes. RNA 11, 241–247. 10.1261/rna.7240905 15701730 PMC1370713

[B3] Bonev B. Mendelson Cohen N. Szabo Q. Fritsch L. Papadopoulos G. L. Lubling Y. (2017). Multiscale 3D genome rewiring during mouse neural development. Cell 171, 557–572. 10.1016/j.cell.2017.09.043 29053968 PMC5651218

[B4] Bouschet T. Dubois E. Reynes C. Kota S. K. Rialle S. Maupetit-Mehouas S. (2017). *In vitro* corticogenesis from embryonic stem cells recapitulates the *in vivo* epigenetic control of imprinted gene expression. Cereb. Cortex 27, 2418–2433. 10.1093/cercor/bhw102 27095822

[B5] Capitano F. Camon J. Licursi V. Ferretti V. Maggi L. Scianni M. (2017). MicroRNA-335-5p modulates spatial memory and hippocampal synaptic plasticity. Neurobiol. Learn. Mem. 139, 63–68. 10.1016/j.nlm.2016.12.019 28039088

[B6] Chen L. Heikkinen L. Wang C. Yang Y. Sun H. Wong G. (2019). Trends in the development of miRNA bioinformatics tools. Brief. Bioinform. 20, 1836–1852. 10.1093/bib/bby054 29982332 PMC7414524

[B7] Chiang H. R. Schoenfeld L. W. Ruby J. G. Auyeung V. C. Spies N. Baek D. (2010). Mammalian microRNAs: Experimental evaluation of novel and previously annotated genes. Genes Dev. 24, 992–1009. 10.1101/gad.1884710 20413612 PMC2867214

[B8] Chien C.-H. Sun Y.-M. Chang W.-C. Chiang-Hsieh P.-Y. Lee T.-Y. Tsai W.-C. (2011). Identifying transcriptional start sites of human microRNAs based on high-throughput sequencing data. Nucleic Acids Res. 39, 9345–9356. 10.1093/nar/gkr604 21821656 PMC3241639

[B9] de Rie D. Abugessaisa I. Alam T. Arner E. Arner P. Ashoor H. (2017). An integrated expression atlas of miRNAs and their promoters in human and mouse. Nat. Biotechnol. 35, 872–878. 10.1038/nbt.3947 28829439 PMC5767576

[B10] DeVeale B. Swindlehurst-Chan J. Blelloch R. (2021). The roles of microRNAs in mouse development. Nat. Rev. Genet. 22, 307–323. 10.1038/s41576-020-00309-5 33452500

[B11] Drobna-Śledzińska M. Maćkowska-Maślak N. Jaksik R. Dąbek P. Witt M. Dawidowska M. (2022). CRISPRi for specific inhibition of miRNA clusters and miRNAs with high sequence homology. Sci. Rep. 12, 6297. 10.1038/s41598-022-10336-3 35428787 PMC9012752

[B12] Eiraku M. Watanabe K. Matsuo-Takasaki M. Kawada M. Yonemura S. Matsumura M. (2008). Self-organized formation of polarized cortical tissues from ESCs and its active manipulation by extrinsic signals. Cell Stem Cell 3, 519–532. 10.1016/j.stem.2008.09.002 18983967

[B13] Fromm B. Billipp T. Peck L. E. Johansen M. Tarver J. E. King B. L. (2015). A uniform system for the annotation of vertebrate microRNA genes and the evolution of the human microRNAome. Annu. Rev. Genet. 49, 213–242. 10.1146/annurev-genet-120213-092023 26473382 PMC4743252

[B61] Fulco C. P. Munschauer M. Anyoha R. Munson G. Grossman S. R. Perez E.-M. (2016). Systematic mapping of functional enhancer–promoter connections with CRISPR interference. Science 354, 769–773. 10.1126/science.aag2445 27708057 PMC5438575

[B14] Gilbert L. A. Larson M. H. Morsut L. Liu Z. Brar G. A. Torres S. E. (2013). CRISPR-mediated modular RNA-guided regulation of transcription in eukaryotes. Cell 154, 442–451. 10.1016/j.cell.2013.06.044 23849981 PMC3770145

[B15] Ha M. Kim V. N. (2014). Regulation of microRNA biogenesis. Nat. Rev. Mol. Cell Biol. 15, 509–524. 10.1038/nrm3838 25027649

[B16] He C. Li Z. Chen P. Huang H. Hurst L. D. Chen J. (2012). Young intragenic miRNAs are less coexpressed with host genes than old ones: Implications of miRNA-host gene coevolution. Nucleic Acids Res. 40, 4002–4012. 10.1093/nar/gkr1312 22238379 PMC3351155

[B17] Hinske L. C. França G. S. Torres H. A. M. Ohara D. T. Lopes-Ramos C. M. Heyn J. (2014). miRIAD—integrating microRNA inter- and intragenic data. Database 2014, bau099. 10.1093/database/bau099 25288656 PMC4186326

[B18] Hiramuki Y. Sato T. Furuta Y. Surani M. A. Sehara-Fujisawa A. (2015). Mest but not MiR-335 affects skeletal muscle growth and regeneration. PLOS ONE 10, e0130436. 10.1371/journal.pone.0130436 26098312 PMC4476715

[B19] Ji L. Bishayee K. Sadra A. Choi S. Choi W. Moon S. (2017). Defective neuronal migration and inhibition of bipolar to multipolar transition of migrating neural cells by Mesoderm-Specific Transcript, Mest, in the developing mouse neocortex. Neuroscience 355, 126–140. 10.1016/j.neuroscience.2017.05.003 28501506

[B20] Jones-Rhoades M. W. Bartel D. P. Bartel B. (2006). MicroRNAs and their regulatory roles in plants. Annu. Rev. Plant Biol. 57, 19–53. 10.1146/annurev.arplant.57.032905.105218 16669754

[B21] Kampmann M. (2018). CRISPRi and CRISPRa screens in mammalian cells for precision biology and medicine. ACS Chem. Biol. 13, 406–416. 10.1021/acschembio.7b00657 29035510 PMC5886776

[B22] Kingston E. R. Bartel D. P. (2019). Global analyses of the dynamics of mammalian microRNA metabolism. Genome Res. 29, 1777–1790. 10.1101/gr.251421.119 31519739 PMC6836734

[B23] Konermann S. Brigham M. D. Trevino A. E. Joung J. Abudayyeh O. O. Barcena C. (2015). Genome-scale transcriptional activation by an engineered CRISPR-Cas9 complex. Nature 517, 583–588. 10.1038/nature14136 25494202 PMC4420636

[B24] Kozomara A. Birgaoanu M. Griffiths-Jones S. (2019). miRBase: from microRNA sequences to function. Nucleic Acids Res. 47, D155–D162. 10.1093/nar/gky1141 30423142 PMC6323917

[B25] Labun K. Montague T. G. Krause M. Torres Cleuren Y. N. Tjeldnes H. Valen E. (2019). CHOPCHOP v3: Expanding the CRISPR web toolbox beyond genome editing. Nucleic Acids Res. 47, W171–W174. 10.1093/nar/gkz365 31106371 PMC6602426

[B26] Le Digarcher A. Lemmers C. Monteil A. Hong C. Varrault A. Bouschet T. (2022). “A CRISPR/Cas9-Based toolkit to test gain- and loss-of-gene function in brain organoids,” in Translational research methods in neurodevelopmental disorders neuromethods. Editors Martin S. Laumonnier F. (Springer US), 75–92. 10.1007/978-1-0716-2569-9_5

[B27] Lee H. B. Sundberg B. N. Sigafoos A. N. Clark K. J. (2016). Genome engineering with TALE and CRISPR systems in neuroscience. Front. Genet. 7, 47. 10.3389/fgene.2016.00047 27092173 PMC4821859

[B28] Lefebvre L. Viville S. Barton S. C. Ishino F. Keverne E. B. Surani M. A. (1998). Abnormal maternal behaviour and growth retardation associated with loss of the imprinted gene Mest. Nat. Genet. 20, 163–169. 10.1038/2464 9771709

[B29] Li K. Liu Y. Cao H. Zhang Y. Gu Z. Liu X. (2020). Interrogation of enhancer function by enhancer-targeting CRISPR epigenetic editing. Nat. Commun. 11, 485. 10.1038/s41467-020-14362-5 31980609 PMC6981169

[B30] Liang Y. Ridzon D. Wong L. Chen C. (2007). Characterization of microRNA expression profiles in normal human tissues. BMC Genomics 8, 166–220. 10.1186/1471-2164-8-166 17565689 PMC1904203

[B31] Lin Y.-L. Mettling C. Portales P. Reynes J. Clot J. Corbeau P. (2002). Cell surface CCR5 density determines the postentry efficiency of R5 HIV-1 infection. Proc. Natl. Acad. Sci. 99, 15590–15595. 10.1073/pnas.242134499 12434015 PMC137761

[B32] Liu S. J. Lim D. A. (2018). Modulating the expression of long non-coding RNAs for functional studies. EMBO Rep. 19, e46955. 10.15252/embr.201846955 30467236 PMC6280642

[B33] Lizio M. Abugessaisa I. Noguchi S. Kondo A. Hasegawa A. Hon C. C. (2019). Update of the FANTOM web resource: Expansion to provide additional transcriptome atlases. Nucleic Acids Res. 47, D752–D758. 10.1093/nar/gky1099 30407557 PMC6323950

[B34] Long L. Guo H. Yao D. Xiong K. Li Y. Liu P. (2015). Regulation of transcriptionally active genes via the catalytically inactive Cas9 in *C. elegans* and *D. rerio* . Cell Res. 25, 638–641. 10.1038/cr.2015.35 25849246 PMC4423076

[B35] Maharry S. E. Walker C. J. Liyanarachchi S. Mehta S. Patel M. Bainazar M. A. (2016). Dissection of the major hematopoietic quantitative trait locus in chromosome 6q23.3 identifies miR-3662 as a player in hematopoiesis and acute myeloid leukemia. Cancer Discov. 6, 1036–1051. 10.1158/2159-8290.CD-16-0023 27354268 PMC5168803

[B36] Marsico A. Huska M. R. Lasserre J. Hu H. Vucicevic D. Musahl A. (2013). PROmiRNA: A new miRNA promoter recognition method uncovers the complex regulation of intronic miRNAs. Genome Biol. 14, R84–R23. 10.1186/gb-2013-14-8-r84 23958307 PMC4053815

[B37] Medley J. C. Panzade G. Zinovyeva A. Y. (2021). microRNA strand selection: Unwinding the rules. WIREs RNA 12, e1627. 10.1002/wrna.1627 32954644 PMC8047885

[B38] Meunier J. Lemoine F. Soumillon M. Liechti A. Weier M. Guschanski K. (2013). Birth and expression evolution of mammalian microRNA genes. Genome Res. 23, 34–45. 10.1101/gr.140269.112 23034410 PMC3530682

[B39] Monteys A. M. Spengler R. M. Wan J. Tecedor L. Lennox K. A. Xing Y. (2010). Structure and activity of putative intronic miRNA promoters. RNA 16, 495–505. 10.1261/rna.1731910 20075166 PMC2822915

[B40] O’Brien J. Hayder H. Zayed Y. Peng C. (2018). Overview of MicroRNA biogenesis, mechanisms of actions, and circulation. Front. Endocrinol. 9, 402. 10.3389/fendo.2018.00402 PMC6085463 30123182

[B41] Ozsolak F. Poling L. L. Wang Z. Liu H. Liu X. S. Roeder R. G. (2008). Chromatin structure analyses identify miRNA promoters. Genes Dev. 22, 3172–3183. 10.1101/gad.1706508 19056895 PMC2593607

[B42] Powrózek T. Krawczyk P. Kowalski D. M. Winiarczyk K. Olszyna-Serementa M. Milanowski J. (2015). Plasma circulating microRNA-944 and microRNA-3662 as potential histologic type-specific early lung cancer biomarkers. Transl. Res. 166, 315–323. 10.1016/j.trsl.2015.05.009 26079400

[B43] Powrózek T. Mlak R. Dziedzic M. Małecka-Massalska T. Sagan D. (2017). Analysis of primary-miRNA-3662 and its mature form may improve detection of the lung adenocarcinoma. J. Cancer Res. Clin. Oncol. 143, 1941–1946. 10.1007/s00432-017-2444-0 28540403 PMC11819407

[B44] Rodriguez A. Griffiths-Jones S. Ashurst J. L. Bradley A. (2004). Identification of mammalian microRNA host genes and transcription units. Genome Res. 14, 1902–1910. 10.1101/gr.2722704 15364901 PMC524413

[B45] Ronchetti D. Lionetti M. Mosca L. Agnelli L. Andronache A. Fabris S. (2008). An integrative genomic approach reveals coordinated expression of intronic miR-335, miR-342, and miR-561 with deregulated host genes in multiple myeloma. BMC Med. Genomics 1, 37. 10.1186/1755-8794-1-37 18700954 PMC2531129

[B46] Schanen B. C. Li X. (2011). Transcriptional regulation of mammalian miRNA genes. Genomics 97, 1–6. 10.1016/j.ygeno.2010.10.005 20977933 PMC3019299

[B47] Seitz H. Royo H. Bortolin M.-L. Lin S.-P. Ferguson-Smith A. C. Cavaillé J. (2004). A large imprinted microRNA gene cluster at the mouse dlk1-gtl2 domain. Genome Res. 14, 1741–1748. 10.1101/gr.2743304 15310658 PMC515320

[B48] Steiman-Shimony A. Shtrikman O. Margalit H. (2018). Assessing the functional association of intronic miRNAs with their host genes. RNA 24, 991–1004. 10.1261/rna.064386.117 29752351 PMC6049507

[B49] Tomé M. López-Romero P. Albo C. Sepúlveda J. C. Fernández-Gutiérrez B. Dopazo A. (2011). miR-335 orchestrates cell proliferation, migration and differentiation in human mesenchymal stem cells. Cell Death Differ. 18, 985–995. 10.1038/cdd.2010.167 21164520 PMC3131940

[B50] Vandesompele J. De Preter K. Pattyn F. Poppe B. Van Roy N. De Paepe A. (2002). Accurate normalization of real-time quantitative RT-PCR data by geometric averaging of multiple internal control genes. Genome Biol. 3, RESEARCH0034. 10.1186/gb-2002-3-7-research0034 12184808 PMC126239

[B51] Varrault A. Eckardt S. Girard B. Le Digarcher A. Sassetti I. Meusnier C. (2018). Mouse parthenogenetic embryonic stem cells with biparental-like expression of imprinted genes generate cortical-like neurons that integrate into the injured adult cerebral cortex. Stem Cells 36, 192–205. 10.1002/stem.2721 29044892 PMC5785436

[B52] Westholm J. O. Lai E. C. (2011). Mirtrons: microRNA biogenesis via splicing. Biochimie 93, 1897–1904. 10.1016/j.biochi.2011.06.017 21712066 PMC3185189

[B53] Xue B. Chuang C.-H. Prosser H. M. Fuziwara C. S. Chan C. Sahasrabudhe N. (2021). miR-200 deficiency promotes lung cancer metastasis by activating Notch signaling in cancer-associated fibroblasts. Genes Dev. 35, 1109–1122. 10.1101/gad.347344.120 34301766 PMC8336896

[B54] Yang D. Lutter D. Burtscher I. Uetzmann L. Theis F. J. Lickert H. (2014). miR-335 promotes mesendodermal lineage segregation and shapes a transcription factor gradient in the endoderm. Development 141, 514–525. 10.1242/dev.104232 24449834

[B55] Yeo N. C. Chavez A. Lance-Byrne A. Chan Y. Menn D. Milanova D. (2018). An enhanced CRISPR repressor for targeted mammalian gene regulation. Nat. Methods 15, 611–616. 10.1038/s41592-018-0048-5 30013045 PMC6129399

[B56] Yi B. Wang S. Wang X. Liu Z. Zhang C. Li M. (2022). CRISPR interference and activation of the microRNA-3662-HBP1 axis control progression of triple-negative breast cancer. Oncogene 41, 268–279. 10.1038/s41388-021-02089-6 34728806 PMC8781987

[B57] Yusa K. Zhou L. Li M. A. Bradley A. Craig N. L. (2011). A hyperactive piggyBac transposase for mammalian applications. Proc. Natl. Acad. Sci. 108, 1531–1536. 10.1073/pnas.1008322108 21205896 PMC3029773

[B58] Zhao Y. Dai Z. Liang Y. Yin M. Ma K. He M. (2014). Sequence-specific inhibition of microRNA via CRISPR/CRISPRi system. Sci. Rep. 4, 3943. 10.1038/srep03943 24487629 PMC3909901

[B59] Zhao J. Ma W. Zhong Y. Deng H. Zhou B. Wu Y. (2021). Transcriptional inhibition of lncRNA gadd7 by CRISPR/dCas9-KRAB protects spermatocyte viability. Front. Mol. Biosci. 8, 652392. 10.3389/fmolb.2021.652392 33778010 PMC7991575

[B60] Zhu L. Chen L. Shi C.-M. Xu G.-F. Xu L.-L. Zhu L.-L. (2014). MiR-335, an adipogenesis-related MicroRNA, is involved in adipose tissue inflammation. Cell Biochem. Biophys. 68, 283–290. 10.1007/s12013-013-9708-3 23801157

