# Supplementary figures and images for: Functional mapping of microRNA promoters with dCas9 fused to transcriptional regulators
Source: Front Genet. 2023 May 5;14:1147222. doi: 10.3389/fgene.2023.1147222 (PMC10196145; doi:10.3389/fgene.2023.1147222)

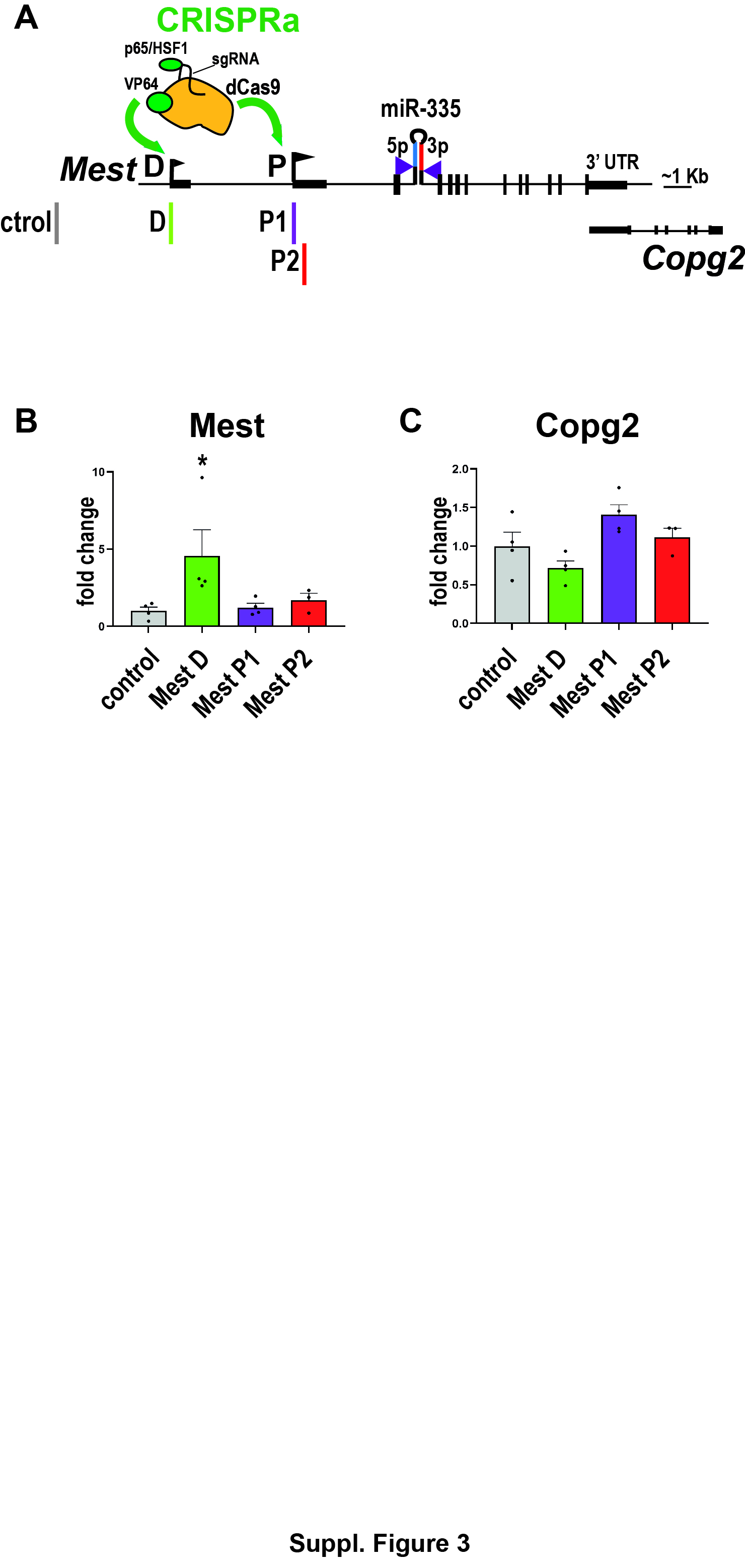

Supplement: Supplementary file 2 [file Image3.JPEG]

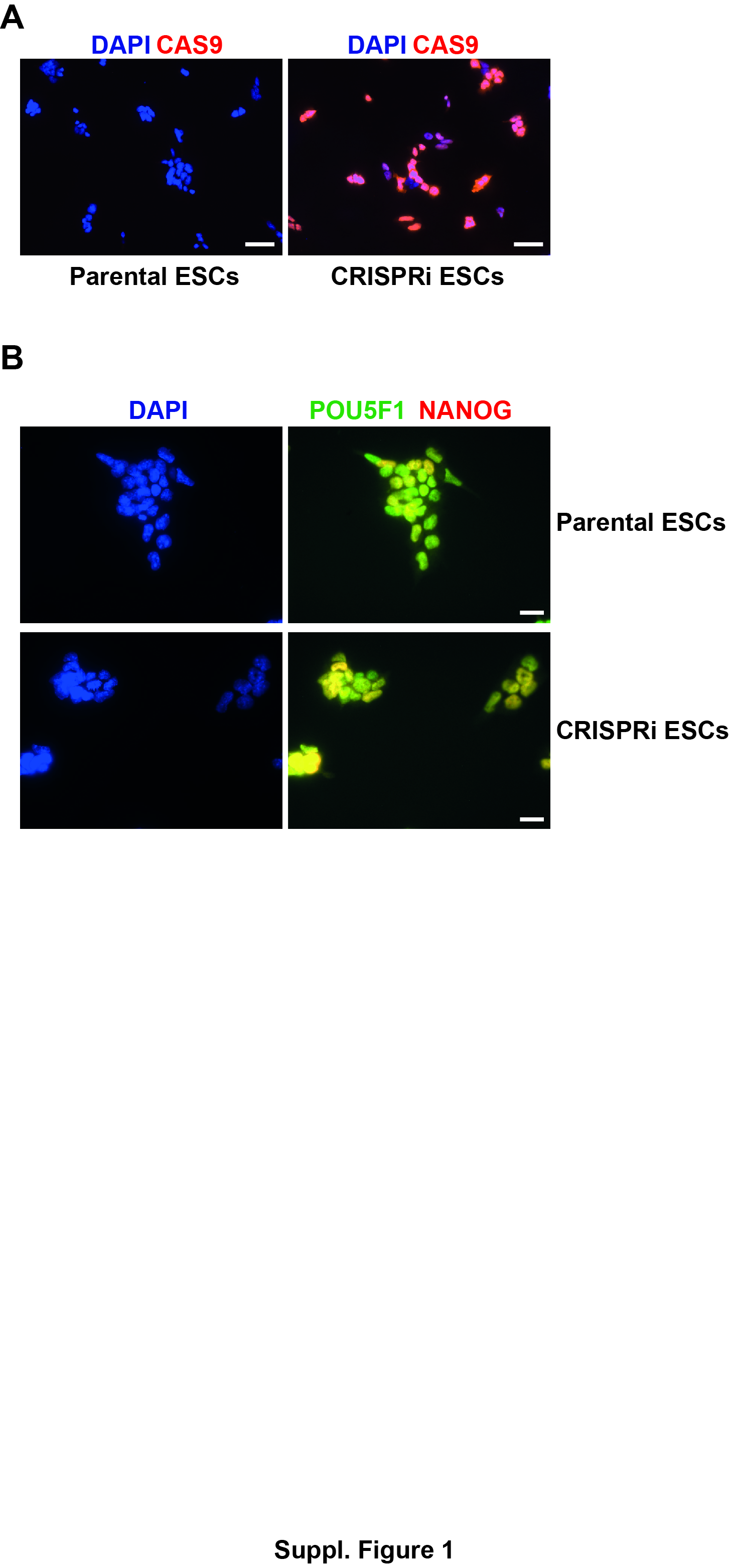

Supplement: Supplementary file 3 [file Image1.JPEG]

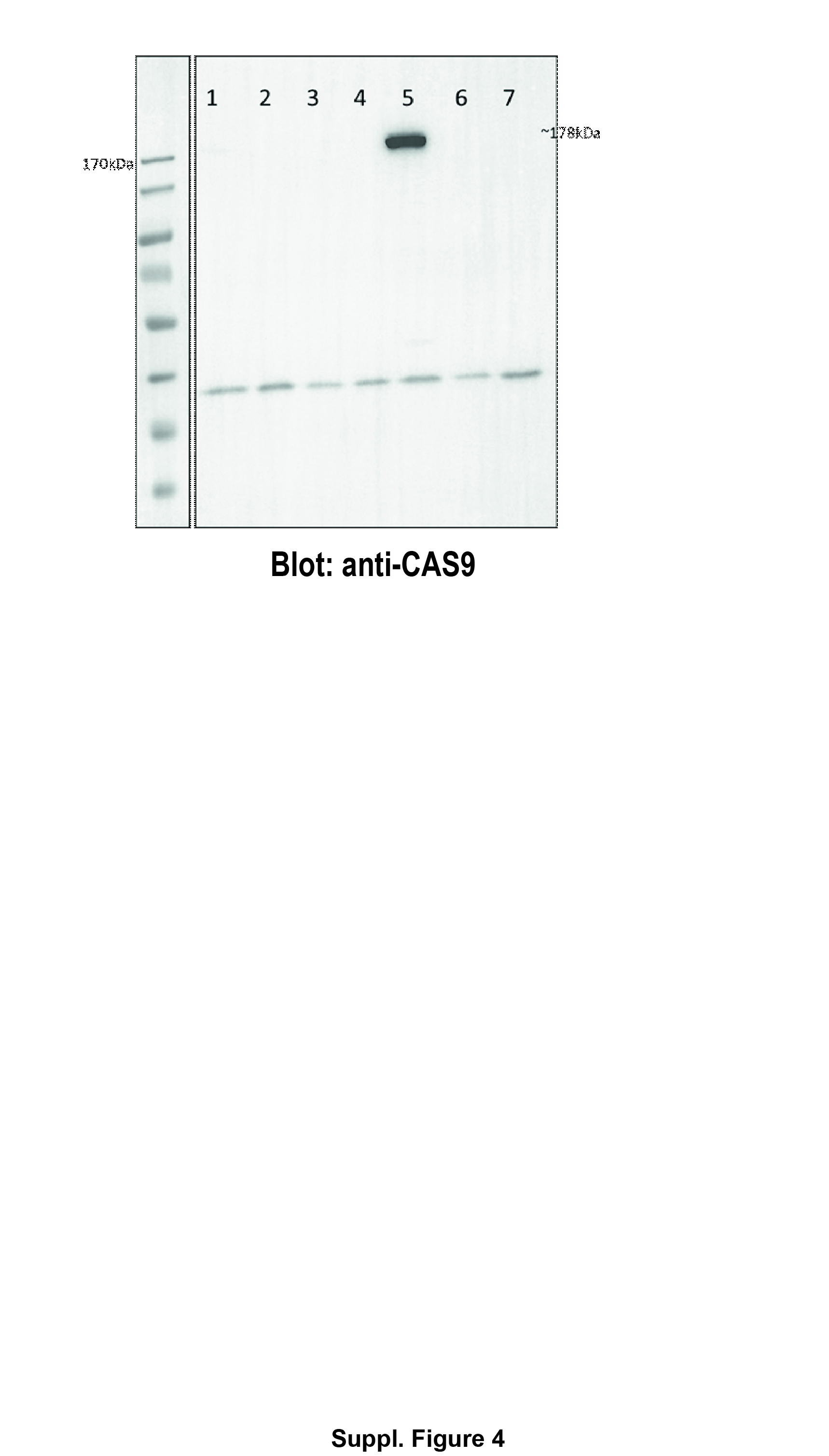

Supplement: Supplementary file 4 [file Image4.JPEG]

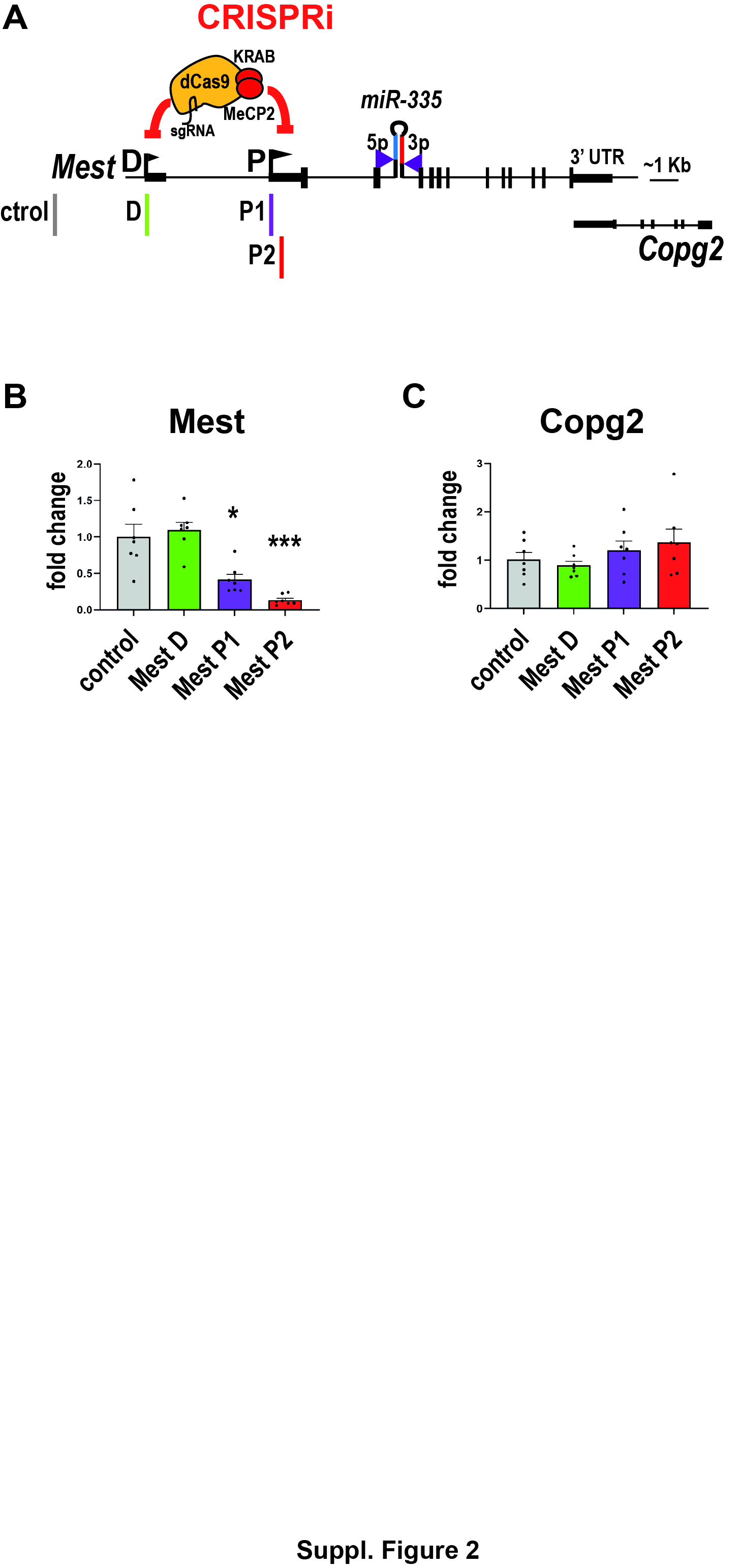

Supplement: Supplementary file 5 [file Image2.JPEG]
