# Supplementary material for: Functional mapping of microRNA promoters with dCas9 fused to transcriptional regulators
Source: Front Genet. 2023 May 5;14:1147222. doi: 10.3389/fgene.2023.1147222 (PMC10196145; doi:10.3389/fgene.2023.1147222)
Supplement: Supplementary file 7 [file DataSheet1.DOCX]

Supplementary Material

Functional mapping of microRNA promoters with dCas9

fused to transcriptional regulators

**Pradeep Kumar^1,2^**^†^**, Mathilde Courtes^3^**^†^**, Céline Lemmers^4^, Anne Le Digarcher^3^, Ilda Coku^3^, Arnaud Monteil^4^, Charles Hong^5,#^, Annie Varrault^1^, Runhua Liu^1,2*^, Lizhong Wang^1,2*^, and Tristan Bouschet^3*^**

*** Correspondence:** <tristan.bouschet@igf.cnrs.fr>

**Supplemental Figure legends**

**Supplemental Figure S1.** Characterization of the dCas9-KRAB-MeCP2 (CRISPRi) ESC line

(A) Immunofluorescence experiments showing the expression of CAS9 in parental E14Tg2a ESCs and their CRISPRi derivatives. Scale bars: 50 µm.

(B) Expression of pluripotency factors POU5F1 (green) and NANOG (red) in parental E14Tg2a ESCs and their CRISPRi derivatives. Scale bars: 20 µm.

**Supplemental Figure S2.** Efficient CRISPRi of *Mest* when targeting its proximal promoter

(A) Structure of mouse *Mest* gene with the CRISPRi module (dCa9-KRAB-MeCP2) directed to either the distal (*D*) (sgRNA D, green) or proximal (P) (sgRNAs P1 -purple- and P2 -red-) promoter of *Mest*. The sgRNA control (grey) has no match in the mouse genome.

(B, C) Repression of the *Mest* proximal promoter downregulates *Mest* expression (B) and does not affect neighbouring *Copg2* expression (C). CRISPRi ESCs were transduced with lentiviruses expressing either sgRNA control, sgRNA D, sgRNA P1, or sgRNA P2. RNAs were measured by RT-qPCR. Data are mean ± sem of seven independent experiments and expressed as fold change over control sgRNA. *: p<0.05, ***: p<0.001 in Mann-Whitney test (comparison with sgRNA control values). Only the sgRNAs targeting the proximal promoter repressed *Mest*.

**Supplemental Figure S3.** Efficient CRISPRa of *Mest* when targeting its distal promoter

(A) Structure of the mouse *Mest* gene with the CRISPRa tool SAM targeting either the distal (sgRNA D, green) or the proximal (sgRNAs P1 -purple- and P2 -red-) promoter of *Mest*. The sgRNA control (grey) has no match in the mouse genome.

(B, C) Transactivation of the *Mest* distal promoter upregulates *Mest* expression (B) and does not affect neighbouring *Copg2* expression (C). CRISPRa SAM ESCs were transduced with lentivirus expressing either SgRNA control, D, P1, or P2. *Mest* (B) and *Copg2* (C) were measured by RT-qPCR. Data are mean ± sem of four independent experiments. *: p<0.1 in Mann-Whitney test. Only the sgRNA targeting the distal promoter upregulated *Mest* RNA.

**Supplemental Figure S4.** Generation of CRISPRa SAM HE293T cells.

Immunoblot analyses of CAS9 protein expression in seven HEK293T colonies. The left panel shows the molecular weight ladder. Only colony #5 (out of 7 colonies) gave a positive signal, at the expected molecular weight (178 kDa), and was selected for further experiments.

**Supplemental Table S1.** Sequences of sgRNAs

**Supplemental Table S2.** Sequences of primers for qPCR
